# Associations between primary motor cortex organization, motor control and sensory tests during the clinical course of low back pain. A protocol for a cross-sectional and longitudinal case-control study

**DOI:** 10.1016/j.conctc.2022.101022

**Published:** 2022-11-03

**Authors:** Sabrine P. Klerx, Sjoerd M. Bruijn, Henri Kiers, Michel W. Coppieters, Jos W.R. Twisk, Annelies L. Pool-Goudzwaard

**Affiliations:** a Faculty of Behavioral and Movement Sciences, Vrije Universiteit Amsterdam, Amsterdam Movement Sciences, Amsterdam, the Netherlands; b Research Group Lifestyle and Health, Section Movement Adaptation and Prognosis, HU University of Applied Sciences, Utrecht, the Netherlands; c Institute of Brain and Behavior, Amsterdam, the Netherlands; d Menzies Health Institute Queensland, Griffith University, Brisbane and Gold Coast, Australia; e Department of Epidemiology and Data Science, Amsterdam University Medical Centre, Amsterdam, the Netherlands; f SOMT University of Physiotherapy, Amersfoort, the Netherlands

**Keywords:** Low back pain, Transcranial magnetic stimulation, Cortical organization, Motor control, Sensory tests, CoG, Center of Gravity, CPM, Conditioned Pain Modulation, EMG, Electromyography, LBP, Low Back Pain, M1, primary motor cortex, MEP, Motor Evoked Potential, MRI, Magnetic Resonance Imaging, MVC, Maximum Voluntary Contraction, PPT, Pressure Pain Threshold, TMS, Transcranial Magnetic Stimulation, QST, Quantitative Sensory Testing

## Abstract

**Background:**

In people with low back pain (LBP), altered motor control has been related to reorganization of the primary motor cortex (M1). Sensory impairments in LBP have also been suggested to be associated with reorganization of M1. Little is known about reorganization of M1 over time in people with LBP, and whether it relates to changes in motor control and sensory impairments and recovery. This study aims to investigate 1) differences in organization of M1 of trunk muscles between people with and without LBP, and whether the organization of M1 relates to motor control and sensory impairments (cross-sectional component) and 2) reorganization of M1 over time and its relation with changes in motor control and sensory impairments and experienced recovery (longitudinal component).

**Methods:**

A case-control study with a cross-sectional and five-week longitudinal component is conducted in participants with LBP (N = 25) and participants without LBP (N = 25). Participants with LBP received usual care physiotherapy. Various tests were administered at baseline and follow-up. Following an anatomical MRI, organization of M1 (Center of Gravity and Area of the cortical representation of trunk muscles) was determined using transcranial magnetic stimulation. Quantitative sensory testing, a spiral-tracking motor control test, graphesthesia, two-point discrimination threshold and various self-reported questionnaires were also assessed. Multivariate multilevel analysis will be used for statistical analysis.

**Conclusion:**

We will address the gaps in knowledge about the association between reorganization of M1 and motor control and sensory tests during the clinical course of LBP. This study is registered at DOI 10.17605/OSF.IO/5C8ZG.

## Highlights

• We assess relations between the organization of M1 and motor and sensory tests.
• This study provides insight in the organization of M1 in LBP in relation to recovery.
• The organization of M1 is assessed via TMS.
• We used whole-brain MRI's for high accuracy of representation of muscles on M1.
• We will use multivariate mixed model analysis to relate M1, motor and sensory tests.

## 1 Introduction

Motor control of trunk muscles is altered in people with low back pain (LBP) [[1], [2], [3], [4], [5]]. Altered trunk motor control in LBP has been related to the (re)organization of the primary motor cortex (M1) [[6], [7], [8]]. Regarding the reorganization of M1 of trunk muscles, a lower level of intracortical inhibition of the lumbar multifidus muscle [9] and a smaller map volume [10] have been observed in people with LBP compared to people without LBP. Furthermore, a relation between map volume and the severity of LBP has been demonstrated [11,12].

To determine the cortical representation, as a measure of cortical reorganization, the location of the Center of Gravity (CoG; the amplitude weighted center of cortical representation of a muscle) and area can be analyzed. The CoG is a valid outcome for measuring this representation [13,14].

There is conflicting evidence regarding the altered location of the CoG of the erector spinae in people with LBP: both a more posterior [10] and a more anterior [11,12] location have been found compared to people without LBP. Additionally, in people with LBP, an increased overlap in the cortical representation of the erector spinae and deep multifidus muscle [10] has been revealed.

Changes over time in the organization of M1 have been shown in people with LBP regarding: increased intracortical inhibition after isometric deep multifidi training [8]; increased intracortical facilitation after repetitive peripheral magnetic stimulation [15]; increased map volume and increased discrete peaks after combined transcranial direct current stimulation and peripheral electrical stimulation compared to a reduced map volume following peripheral electrical stimulation alone, and no difference in the amount of peaks following either one of the stimulations separately [16]. A prospective cohort study revealed an association between a smaller map volume of M1 in the acute stage of LBP with a higher pain intensity at 6-month follow-up. However, after adjustment for confounding predisposing factors, such as blood biomarkers for inflammation, psychological variables (emotional and cognitive pain experience) and mechanical pain sensitization, this association was no longer significant [17].

Little is known about changes of the CoG of trunk muscles over time in people with LBP, and how this relates to recovery. A proof-of-principle study revealed an anterior and medial shift of the CoG of the transversus abdominis muscle following exercises of contraction of this muscle, whereas in the control intervention of self-paced walking no changes were observed [7]. In addition, a prospective cohort study revealed that a smaller L3 map volume was a predictor for LBP at 6-months follow-up, in contrast to the CoG which revealed not to be a predictor [18].

Sensory impairments are also suggested to be associated with reorganization of M1 [3,19]. Adaptive motor behavior in people with LBP may lead to selective muscle activation and kinematics. This will lead to a change in sensory feedback from the lumbar area. This, in turn, might contribute to neuroplastic changes in the motor and sensor cortex and to proprioceptive impairments reducing the ability to control lumbar movement [3]. This suggested relation has hardly been studied, and has not yet been demonstrated [12].

Because of the scarce and inconsistent evidence, there is a need for a better understanding of the relations between motor control changes, sensory changes, and M1 reorganization of trunk muscles in people with LBP. To investigate this relation, we will conduct a case-control study with both a cross-sectional and longitudinal component. The aim of the cross-sectional part is to investigate differences between people with LBP and without LBP regarding the reorganization of M1 of trunk muscles, and the performance on motor control and sensory tests. In addition, the association between the organization of M1 and the performance on motor control and sensory tests will be studied. The aim of the longitudinal part is to analyze reorganization of M1 of trunk muscles as well as changes in motor control and sensory test performance over time, within and between people with LBP (recovered and non-recovered) and without LBP. In addition, the associations between reorganization of M1, and changes in motor control and sensory test performance over time, and between groups, will be studied.

## 2 Methods

### 2.1 Study design

A case-control study with a cross-sectional component and five-week longitudinal component was conducted in participants with and without LBP. All participants provided written informed consent before participating in the trial. The study has been approved by the Medical Ethics Committee Brabant, number NL70934.028.19/P1944. The study is registered in the Open Science Framework (10.17605/OSF.IO/5C8ZG). The STROBE checklist for reporting observational studies has been followed.

### 2.2 Participants

We included people with LBP (>24 h) [20] who were seeking medical or physiotherapy care, who either had chronic LBP (pain duration of >12 weeks) and had recently experienced a flare up, or people with recurrent LBP. LBP was defined as pain between the lower rib margins and the buttock creases [21]. A flare up was defined as an increase in pain of at least two points in pain intensity on a 0–10 Numeric Pain Rating Scale and also an increase in the impact of LBP on usual activity [22]. Recurrent LBP was defined as an episode of LBP lasting >24 h, separated by a pain-free period of more than one month without LBP [23], and where LBP had an impact on usual activity. With these selection criteria, we aimed to include people who were anticipated to experience fluctuations or recovery in pain and function. The following exclusion criteria were used: major spinal pathology, a history of lumbar radiculopathy or spinal operation, circulatory diseases, or pregnancy and the six-month postpartum period, younger than 18 or older than 65 years of age, or not meeting the safety criteria for Magnetic Resonance Imaging (MRI) or Transcranial Magnetic Stimulation (TMS) [24]. People without LBP had to be free of an episode of LBP in the preceding three years and were matched for sex and age to the people with LBP. People with LBP were recruited from five primary care physiotherapy clinics in The Netherlands. People without LBP were recruited from the acquaintances, friends, or relatives of the patients.

### 2.3 Procedure

**Fig. 1:**
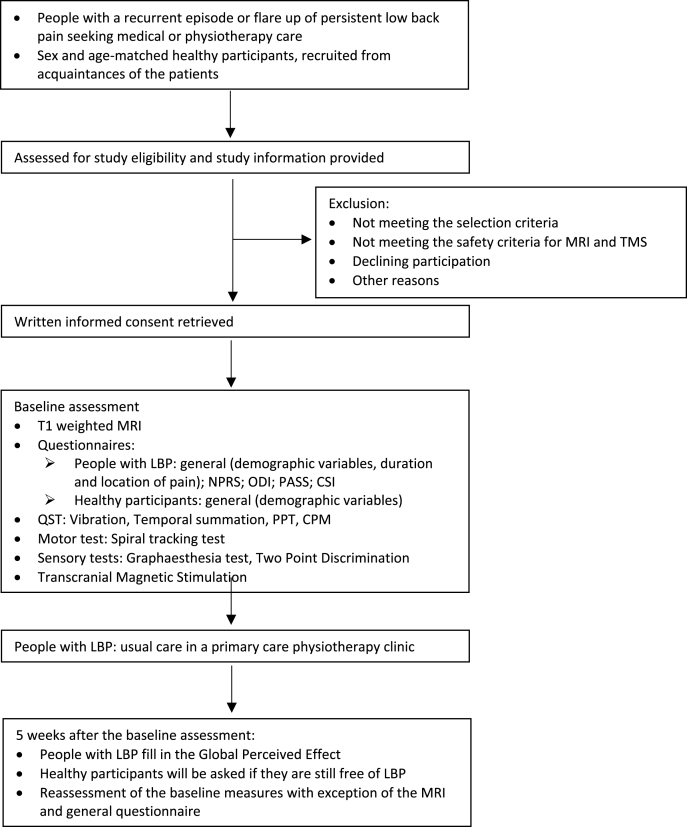
Flowchart of the study. MRI, Magnetic Resonance Imaging; TMS; Transcranial Magnetic Stimulation; LBP, Low Back Pain; NPRS, Numeric Pain Rating Scale; ODI, Oswestry Disability Index; PASS, Pain Anxiety Symptom Scale; CSI, Central Sensitization Inventory; PPT, Pressure Pain Threshold; CPM, Conditioned Pain Modulation.

All participants were measured twice with an interval of five weeks (± one week), since a marked improvement is expected in the first six weeks of a LBP episode [25]. During this five-week interval, the participants with LBP received usual care in a primary care physiotherapy clinic. The therapists of these clinics received information about possible exercises for motor control and sensory accuracy, but were free in their choice of therapy form, duration, and intensity. However, during therapy they were not allowed to use the tests of which the results served as outcomes in the present study. All participants first underwent a T1 weighted MRI of the brain, after which they completed self-reported questionnaires, performed quantitative sensory tests, a motor control test, sensory tests and underwent TMS to determine the organization of M1. The MRI was only conducted before the first measurement. Below, each of these assessments is described in detail. See Fig. 1 for a flowchart of the study.

### 2.4 Assessments

#### 2.4.1 Questionnaires

All participants completed a questionnaire regarding demographic variables. Participants with LBP also completed 1) a general questionnaire containing questions on location and duration of their pain; 2) a Numeric Pain Rating Scale (0–10); 3) the Oswestry Disability Index; 4) the Pain Anxiety Symptom Scale; and 5) the Central Sensitization Inventory. All questionnaires have adequate reliability and validity [[26], [27], [28], [29], [30]]. At 5-week follow-up, participants with LBP indicated their overall level of recovery on a Global Perceived Effect scale (GPE-Dutch Version, 7-point Likert scale) and completed the Numeric Pain Rating Scale, Oswestry Disability Index, Pain Anxiety Symptom Scale and Central Sensitization Inventory. Participants without LBP reported whether they were still free of LBP.

For our analyses, we are interested in those participants with LBP who showed recovery, versus those who did not. These groups (recovered and non-recovered LBP participants) are defined based on their ‘Global Perceived Effect’ after 5 weeks. The LBP participants is asked how much their back condition has improved or deteriorated on a 7-point Likert scale. Category 1 (‘completely recovered’) and category 2 (‘much improved’) will be considered as recovered. Category 3 (‘slightly improved’), 4 (‘equal’) and 5 (‘slightly deteriorated’) will be considered as ‘unchanged’, category 6 (‘much deteriorated’) and 7 (‘completely deteriorated’) as deteriorated. Therefore, category 3 till 7 will be considered as non-recovered [31].

#### 2.4.2 Quantitative sensory testing

Vibration sense, pressure pain threshold (PPT), conditioned pain modulation (CPM) and temporal summation of pain were performed according to the recommended protocols [32,33]. For these tests, the participant was positioned prone, and familiarization was performed on the right hand.

1) Vibration sense

A Rydel-Seiffer tuning fork (64 Hz, 8/8 scale; US Neurologicals, WA) was percussed and then placed over the spinous process of L4. When the participant indicated that vibration could no longer be felt, the corresponding value was determined from the scale on the prongs of the fork, ranging from 0 to 8 (with higher scores better vibration sense). The mean of three consecutive trials is used to determine the vibration threshold [[32], [33], [34]].

2) Temporal summation of pain

First, a single pinprick (256 mN, MRC-systems GmbH, Heidelberg, Germany) was applied on the skin, 3 cm lateral to the spinous process of L5 on the (most) painful side. After 10 s, a train of 10 stimuli was applied at 1 Hz within an area of 1 cm^2^. This was repeated five times, with a 1-min rest between repetitions. Temporal summation was calculated by subtracting the mean Numeric Pain Rating Scale of the single stimuli from the Numeric Pain Rating Scale of the trains of 10 stimuli.

3) Pressure Pain Threshold

A digital algometer (Wagner Instruments Model FDX-25, Greenwich, USA) was placed perpendicular to the skin, 3 cm lateral to the L5 spinous process on the (most) painful side, as outlined for the temporal summation. In case of equal intensity of LBP on both sides, the side of the measurement was randomized. The pressure was increased at a rate of ∼5 N/s. The participant was asked to indicate when the sensation of pressure changes to a sensation of painful pressure. The threshold is based on the mean of three repetitions.

4) Conditioned Pain Modulation

After three baseline PPT measures, the participant submerged the right hand in a portable insulated 10 L container (Curver, the Netherlands), filled with water cooled to 10° Celsius, as measured with a digital thermometer). As soon as the pain intensity for the hand in the water reached 4/10, three PPT measures were performed at the same location as the baseline PPT measures, with 30s rest periods between PPTs. Numeric Pain Rating Scale scores for hand pain were recorded following each PPT. The relative and absolute CPM effect will be calculated as recommended [35]. The relative CPM effect will be calculated by subtracting the PPT value obtained during the cold pressor test from the baseline PPT; then the resultant value is divided by the baseline PPT. The value is then multiplied by 100 to obtain percentages. The absolute CPM effect will be calculated by subtracting the mean of the three PTT measures during the conditioning stimulus from the baseline PPTs [36]. In this way, pain inhibition will be reported by a negative value and pain facilitation by a positive value [35].

#### 2.4.3 Motor control

Motor control was assessed with a spiral tracking test based on a previous study [4]. A custom-built sensor was attached to the skin at the level of the spinous process of T12. The sensor consists of an inertial measurement unit (MPU9250) and a microcontroller that processes the measurements with a sample rate of 100 Hz (SAMD21G18A). The orientation of the movement sensor was visualized by a green pointer on a computer monitor, placed in front of the participant. The participant was sitting in a relaxed upright position (i.e., not slumped, not in lordosis). The monitor also showed a red pointer (target) and a spiral. The red pointer started moving anticlockwise along the lines of the spiral figure, from the center to the periphery. The task for the participant was to follow the red pointer as precise as possible by moving the green pointer with the torso. The test ended automatically when the red pointer reached the end of the spiral. The test took about 2 min to complete.

The tracking error will be calculated based on the absolute difference between the target angle and the actual inclination angle of the trunk in the sagittal (x) and transversal (y) axis of motion [4]. In a separate reliability study, we will determine the variables that are most suitable for further analysis. Potential outcome variables will be selected from three categories: 1) degrees (the mean of a subset of the tracking errors, ranging from the closest 10% to the closest 90% errors; 2) time (the mean of a subset of the tracking errors, expressed in the time spent in an area smaller than X° of error) and 3) path (the sum of all errors, in degrees over one trial). More information about the measurement instrument and procedure is provided in Appendix A. Data from the sensors will be analyzed using custom-written Matlab scripts (R2014B, The MathWorks, Natick, MA).

#### 2.4.4 Sensory tests

1) Graphaesthesia test

After a video instruction for the participant about the test procedures, a cross was drawn on the lower back, creating four quadrants of ∼5 × 5centimeters, with L3 at the center of the cross. Five numbers were drawn per quadrant (in total 20) with the back of the holder of a monofilament [37]. The participant had to recognize the number within 3 s. Numbers between zero to nine were used, apart from one and five as those are too easy to identify. The numbers were randomized per quadrant. Familiarization was performed by drawing one random number in each quadrant. The participant received no feedback on whether the number was correctly indicated during the familiarization and test. At the five-week follow-up, the same numbers were used in the same quadrant as at baseline, but in a different order. The error rate will be calculated by dividing the number of incorrect answers by 20.

2) Two-point discrimination threshold

After a video instruction for the participant about the test procedures, two-point discrimination threshold testing was performed at 2 cm left and right from the spinous process of L1, L3 and L5 (six locations) using a 2-point discriminator (Carolina Biological Supply Company, Burlington, NC, USA). The calipers rest for 1 s perpendicular on the skin with a pressure corresponding to first blanching of the skin [38]. The test started with the calipers at 20 mm distance which was increased in five mm steps as long as the participant identified only one stimulus. When the participant identified two stimuli, the caliper was applied again with another 5 mm step increase. If the participant again identified two points, the distance when two stimuli were identified first is considered as the 2-point discrimination threshold [38]. The sequence of the location was randomized per measurement session. The same sequence was used for every participant. To keep the participant unaware of the increase in distance between the two points of stimulation, once per five stimuli only one point or two points with the biggest distance of the 2-point discriminator were stimulated [38]. These ‘in between’ stimulations were not used for data analysis. Familiarization was performed by placing the calipers once over three locations (L1, L3 and L5) left or right). The locations and distances of the calipers for familiarization were randomized. The participant received no feedback on the correctness of feeling one or two points during the familiarization and test.

#### 2.4.5 Reorganization of M1

For more precise navigation of TMS, and to enable analysis of the same positions over time [39], we performed T1 weighted MRI scans for each participant (SIEMENS MAGNETOM Vida-XQ-32 Numaris/X VA20A-04 ML; matrix size 263mmx 350 mm x 350 mm, Voxel Size 1.0 × 1.0 × 1.0 mm³, TR/TE 2400/2.26 ms). Whole-brain grey matter was segmented using SPM12 software [40] for use in neural navigation (Neural Navigator 3.4, BrainScience Tools, The Netherlands).

Surface electromyography (EMG) was used bilaterally to record muscle activity of the longissimus muscle at the level of L3 and L5, and of the internal and external abdominal oblique muscles. Disposable, bipolar pre-gelled rectangular surface ECG electrodes (Ambu Blue Sensor N, Medicotest, Ølstykke, Denmark: AG/AgCl) were used. These electrodes were placed pairwise according to the SENIAM recommendations [41], after cleaning the skin with alcohol. The ground electrode was attached to the skin at the level of the spinous process C7. EMG was captured during the full TMS stimulation protocol at a rate of 2 kHz using a 16-channel Porti EMG device (Twente Medical Systems International B.V., Enschede, The Netherlands).

Prior to the TMS measurements, the MVC was determined. The participant was asked to perform a maximal contraction against resistance in sitting for 5 s and to do so three times [11]. To lower the threshold at which a Motor Evoked Potential (MEP) could be evoked, pre-activation at 20% of the Maximum Voluntary Contraction (MVC) of the longissimus muscle was asked during the TMS measurements. The pre-activation percentage of 20% was based on previous research [10,12,16,17,42], and did not cause subjective signs of fatigue in any of the participants.

This activation was obtained by having the participant sitting with a straight back, and when required resist a weight attached via a pully system to a custom made velcro harness which pulls the participant forward. On a computer screen a feedback line was provided based on the % of MVC the participant was using. A small deviation of the feedback line was allowed [12]. The tester gave verbal instructions when the deviation of the feedback line was too large, e.g. when the participant sat in a slumped position.

A Magstim 200^2^ stimulator (Magstim Company Ltd., Whitland, Dyfed, UK) was used to deliver a single-pulse TMS. The hemisphere contralateral to the side of the highest pain intensity was stimulated, using a figure-of-eight coil with 70 mm windings. The coil was orientated 45° to the sagittal plane, tangential to the scalp to induce currents in the cortex along the posterior to anterior direction [43]. Neural navigation software stored the position and orientation of the coil with respect to the head for each stimulation. The stimulation intensity was set at 100% output of the stimulator, as 120% of the motor threshold of the longissimus muscle generally exceeded the maximum output of the stimulator for most participants [9,10]. The hemispheres in the participants without LBP were measured corresponding to the hemispheres measured in the LBP participants.

During the TMS protocol, the EMG activity of the longissimus muscle at the level L3 of the painful site was monitored for the presence of MEPs. We used a protocol with 100 stimulations at pseudorandom positions using an interstimulus interval of approximately 4 s [42,44], covering the area of the motor cortex using a predefined 7 × 7cm grid, starting at the midline of the vertex. When we also found MEPs at the borders of the grid, stimulations were given in the surroundings, to make sure the area that can be stimulated was completely covered. The measurements were conducted according to the TMS checklist for methodological quality [45]. The MEPs of the other muscles were also recorded, but these were not monitored online. These data will also be reported and analyzed.

The data of the neural navigation, combined with the EMG will be analyzed in Matlab (R2019b) in accordance with Jin et al., 2022 [43]. The EMG data will be high pass filtered at 30 Hz. First, to correct for differences in the shape and the size of an individual's brain, registration to Montreal Neurological Institute space will take place [46]. Then the MEPs will be defined as 500 ms episodes following a stimulation in which the peak-to-peak amplitude is higher than 20 × σ. MEPs with peak-to-peak amplitude higher than 1 mV will be removed as these are likely artefacts. We will visually inspect all identified MEPs. Then, we will calculate for each muscle (longissimus muscle level L3 and L5, internal and external abdominal oblique muscles a) the CoG [10] and b) the cortical area from which stimulations can be elicited, by means of our custom algorithm (see Jin et al., 2022 [43] and https://github.com/marlow17/surfaceanalysis), see Appendix B).

### 2.5 Sample size estimation

The sample size was calculated based on a cross-sectional comparison between two groups, using a continuous outcome, with a power of 0.80 and an α of 0.05. We used the mean and pooled standard deviation (0.68) regarding the CoG of the longissimus muscle from previous similar research, investigating the differences in CoG between the groups participants with LBP (n = 27; mean (SD): 1.4 (0.61)) and participants without LBP (n = 23; mean (SD:) 0.8 (0.77)) [9]. The calculation produced a required sample size of N = 21 per group. Taking a 15–20% drop out risk into account, the required sample size per group was 25.

### 2.6 Statistical analysis

Missing data will be addressed according the STROBE statement [47]. When data are missing <5% per variable per case, complete case analyses will be performed. The type of missing data will be first checked and reported. When necessary, multiple imputation will be performed before analysis.

To analyze cross-sectional differences between participants with and without LBP regarding the organization of M1 of trunk muscles and the performance on motor control and sensory tests, multivariate mixed model analyses will be used, with a 2-level structure. Regarding the organization of M1 of trunk muscles, the MEPs (i.e., longissimus at level L3 and L5, internal and external abdominal oblique muscles) are clustered within the participant. Regarding the motor control and sensory tests, the test scores are clustered within the participant. The same analyses will be used to analyze the association between the organization of M1 of trunk muscles and the performance on a motor control and sensory tests. For the latter, the organization of M1 of trunk muscles will be used as outcome.

To analyze changes over time in organization of M1 of trunk muscles and to analyze differences in changes over time between participants with LBP (recovered and non-recovered) and participants without LBP, multivariate mixed model analyses with a 3-level structure will be used. The MEPs are clustered within the repeated measurements and the repeated measurements are clustered within the participant. The same analytical procedure will be followed to analyze the differences in changes over time in performance on motor control and sensory tests between participants with LBP (recovered and non-recovered) and participants without LBP. In these multivariate mixed model analyses, time, group and the interaction between time and group will be added.

Finally, the longitudinal associations between organization of M1, and the motor control and sensory tests performance over time, will be analyzed with multivariate mixed model analyses. For the latter, again, the organization of M1 of trunk muscles will be used as outcome.

## 3 Discussion

This study will provide evidence about 1) differences in the organization of M1 of trunk muscles between people with and without LBP, and whether the organization of M1 relates to motor control and sensory impairments (cross-sectional component) and 2) reorganization of M1 over time and its relation with changes in motor control and sensory impairments and experienced recovery (longitudinal component).

In this study we applied the following procedures to facilitate a precise navigation and calculation of the organization of M1: 1) we used individual whole-brain anatomical MRI's for the navigation of TMS. This makes it possible to adjust for differences in the shape and the size of the participants' brain and to enable analysis of the same absolute positions over time; 2) in the data-analysis, a custom-made and novel 3D analysis method will be used to calculate the representations of muscles on M1. This method allows calculating the cortical area from which a muscle can be excited, while explicitly excluding stimulations where no MEP was found, and without making assumption about the underlying geometry; 3) since we use a pseudo-random stimulation protocol, our stimulations are closer together than grid-based protocol studies, thus allowing for greater spatial resolution of our outcome measures [44].

The representation of several trunk muscles on M1 will be calculated, and subsequently a possible overlap of these muscles can be demonstrated. This could provide us with more insight into the theory of loss of differential activation of trunk muscles in LBP [10]. Because we have multiple outcomes and variables in this study, we find it appropriate to carry out a multivariate multilevel analysis, where we can correct for multiple testing and dependency of observations.

This study has some limitations. We defined the selection criteria in such a way that we anticipated a proportion of the participants to recover after five weeks. We therefore did not want to solely recruit people with persistent pain, but opted to include people with a fluctuating pain trajectory. However, it is possible that either the number of recovered or the number of non-recovered LBP participants will be small. This may influence the power of the statistical analysis. However, in general the power of longitudinal analyses is higher than the power of cross-sectional analyses, because of the gain in the number of observations. Despite some loss because of the partly correlated observations, more observations will remain for the analysis than in a cross-sectional design. Based on the sample size calculation, for the comparison between participants with LBP and participants without LBP of cortical reorganization over time, the power is therefore expected to be sufficient.

Although people with longer lasting symptoms might be more prone to reorganization of M1, we decided to include participants with a pain duration >24 h [20] who were seeking medical or physiotherapy care, since a marked improvement is expected in the first six weeks of a LBP episode [25].

In this study we used 100% of the maximum stimulator output, as 120% of the motor threshold of the longissimus muscle generally exceeds the maximum output of the stimulator [9,10]. Therefore, no individual stimulation intensity was used. Subsequently, for some participants the stimulation intensity could be too high for accurate determination of the area [43] and for others too low for inducing MEPs.

We analyze the CoG and area, two valid measurement outcomes indicating the representation of trunk muscles on M1. We will not analyze several other outcomes regarding cortical reorganization as intracortical inhibition and facilitation, map volume and discrete peaks, since this requires a different methodology. Silent period will exploratory be analyzed in a subanalysis. Therefore, no conclusions can be drawn regarding the outcomes: intracortical inhibition and facilitation, map volume and discrete peaks. We expect that this study provides information about the relation of reorganization of M1 with clinical motor control and sensory tests performance and recovery of LBP. This could help to further develop theories about the interaction between reorganization of M1 and changes in motor control and sensory performance during the clinical course of LBP [3,19].

## Authors’ contributions

SK, AP, HK and MC designed the study. SK, SB and AP designed the TMS procedure. SK and JT developed the statistical plan. All authors critically read the various drafts of the manuscript and approved the final version of the manuscript.

## Declaration of competing interest

None declared.

## Data Availability

No data was used for the research described in the article.
