# Supplementary material for: Associations between primary motor cortex organization, motor control and sensory tests during the clinical course of low back pain. A protocol for a cross-sectional and longitudinal case-control study
Source: Contemp Clin Trials Commun. 2022 Nov 3;30:101022. doi: 10.1016/j.conctc.2022.101022 (PMC9647172; doi:10.1016/j.conctc.2022.101022)
Supplement: Multimedia component 1 [file mmc1.docx]

**Appendix A. Motor control test; Spiral tracking test**

Measurement instrument

The measurement instrument consists of a sensor with two components: an inertial measurement unit (MPU9250) and a microcontroller that processed the measurements with a sample rate of 100 Hz (SAMD21G18A). The inertial measurement unit consisted of a three-axis accelerometer, a magnetometer and a gyroscope with a Digital Motion Processor (DMP). The sensor readings are fed to a Direction Cosine Matrix (DCM) algorithm, which provides a complete measurement of the orientation.

Procedure

In the Spiral Tracking Test, a fixation belt was fitted over the anterior superior iliac spines and fixated to a weight rack (Figure A.1). The participant was instructed to keep the pressure of the fixation belt as constant as possible during the test. Two rolls were placed between the pelvis and the weight standard. This setup was chosen with the purpose of realizing the participant moving the trunk as a whole instead of moving the lumbar spine into lordosis and kyphosis. We designed a low-tech setup which could be used in a typical physiotherapy setting.

The sensor was attached at the level of spinous vertebrae process thoracic 12 using ECG foam electrodes (Amstel Medical, Amstelveen, the Netherlands, with a diameter of 55mm; Droh, Mainz, Germany). The signal of the movement sensor was visualized by a green point on a computer monitor, placed in front of the participant. The green point was located at the center of the screen when the participant sat in a comfortable neutral position. The spiral comprises 13,5 x 13,5 degrees. The monitor also showed a spiral with a red point on the spiral. At the start of the test the red point started moving anticlockwise along the lines of the spiral figure, from the center to the periphery. The task for the participant was to trace the red point as precise as possible with the green point that represented the orientation of the accelerometer. The test ended automatically when the red point had reached the end of the spiral. The test took about two minutes to complete.

Before starting the trial, the participant was allowed to familiarize for two minutes. The familiarization consisted of moving the green point up, down, left and right two times on the monitor and of one attempt to follow the spiral, without following the red point, with the green point by moving the torso. Unlike during the actual test, the researcher provided feedback regarding their performance during the familiarization period. The clinical set-up is shown in Figure A.1 and a visual presentation of the measurement outcome on the spiral is shown in Figure A.2.


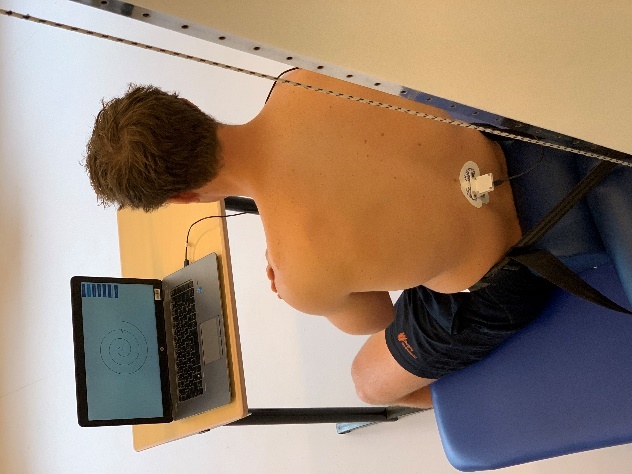


Figure A.1 Clinical set-up Spiral Tracking Test


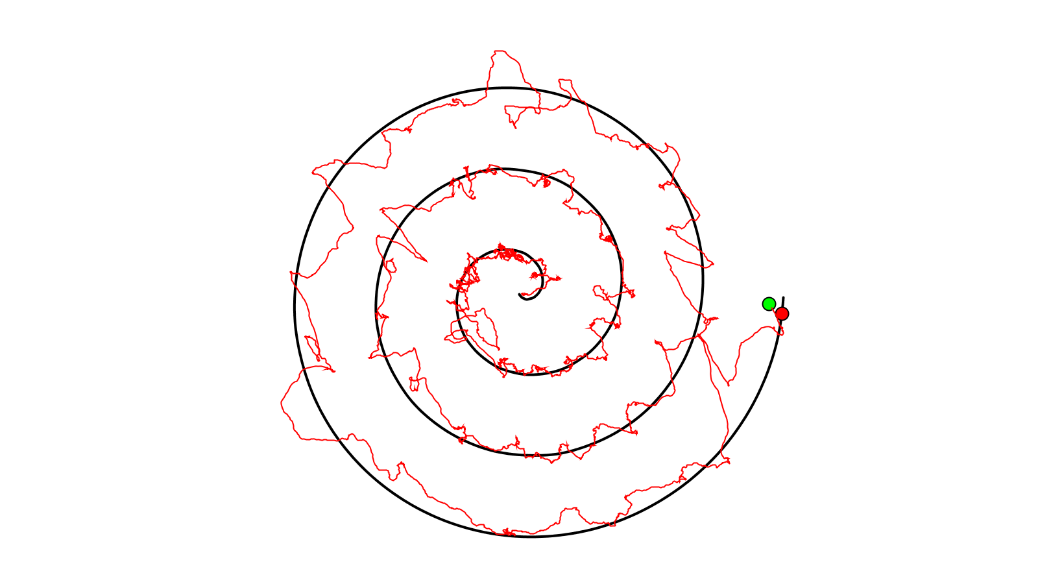


Figure A.2 Example of the Spiral Tracking Test

**Appendix B. Data analysis Organization of M1**

The following description is also overlapping with the supplementary materials found in Jin et al. 2021 (1). For correction for differences in the shape and the size of an individuals’ brain, registration to Montreal Neurological Institute space will take place.

First, we will calculate the mean value μ and the standard deviation σ using the baseline value of signals (-100 ms to -1 ms before stimulus). Then we select the amplitude peaks in the epoch 5 ms to 100 ms after stimulus. Peaks are encountered if the peaks are μ±2∙σ. The latency will be set as the first sample after stimulus at which the signal exceeds μ+2∙σ if the first peak is a maximum. Meanwhile, we set the MEP offset as the first sample after the second peak at which the signal is larger than μ-2∙σ (if the second peak is a minimum) or smaller than μ+2∙σ (if the second peak is a maximum). To select the MEP, first, we define that the peak-to-peak amplitude has to be higher than 20×σ. Then, we will remove the EMG whose peak-to-peak amplitude is higher than 10mV to remove the remaining artifacts (probably due to bad electrode contacts or broken cables).

Area definition

We will first project the (proper≠NaN amplitude) stimulation points to the nearest surface vertices to define the area. Then we select a ‘search area’ via the minimum and maximum 3D coordinates of these vertices and yields projected points. We will convert the mesh into a weighted graph with weights defined as the Euclidean distance between vertices. The shortest connecting paths between all pairs of points will be searched using Dijkstra’s algorithm (2). Finally, we add all vertices on the paths to the point set and repeat the searching until the number of points no longer changes. We select the vertices in the region of primary motor cortex (‘precentral L’ in the atlas Mindboggle6). The area size in the cortex will be computed via Heron’s formula (the areas are triangulated). We will also calculate the CoG (the amplitude weighted center of the map) for each muscle (longissimus muscle at level L3 and L5, internal and external abdominal oblique muscles) using the formula: CoG=Σ(Vi x Xi) /Σvi ; Σ(Vi x Yi)/Σvi, ; Σ(Vi x Zi)/Σvi, where: Vi= MEP amplitude at site I, which has the the coordinates Xi,Yi, Zi (3).

Since the area and CoG are measures that are dependent on the amount of stimulations included in the calculation, it is important that the amount of elicited MEPs does not vary wildly over subjects and time-points. We will include so many stimulations that 75 to 80% of the cortex stimulations per participant remain.

**References**

1. Jin F, Bruijn S, Daffertshofer A. A new protocol for multiple muscle mapping using nTMS. biorxiv. 2021;July 30.

2. Dijkstra EW. A note on two problems in connexion with graphs. Numer Math. 1959;1(1):269–71.

3. Tsao H, Danneels LA, Hodges PW. ISSLS prize winner: Smudging the motor brain in young adults with recurrent low back pain. Spine (Phila Pa 1976). 2011 Oct 1;36(21):1721–7.
